# Designing Effective Antimicrobial Nanostructured Surfaces: Highlighting the Lack of Consensus in the Literature

**DOI:** 10.1021/acsomega.2c08068

**Published:** 2023-04-20

**Authors:** Thomas E. Catley, Rebecca M. Corrigan, Andrew J. Parnell

**Affiliations:** † Department of Physics and Astronomy, University of Sheffield, Hicks Building, Hounsfield Road, Sheffield S3 7RH, United Kingdom; ‡ Molecular Microbiology, School of Biosciences, University of Sheffield, Firth Court, Sheffield S10 2TN, United Kingdom

## Abstract

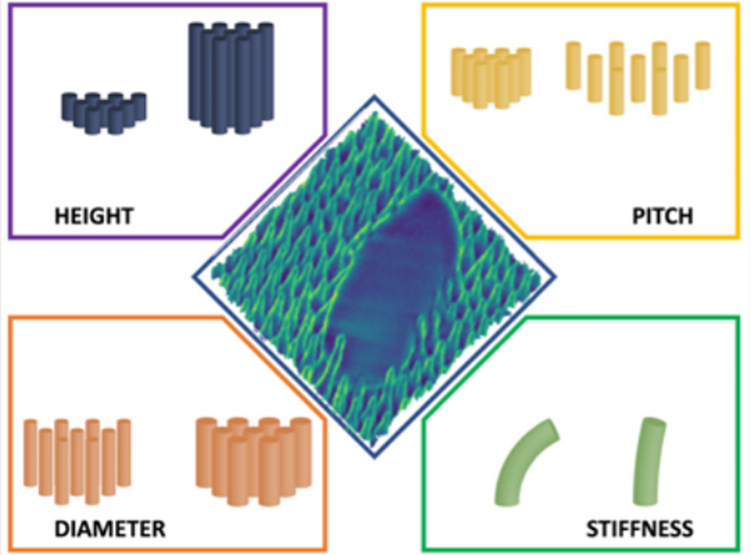

Research into nanostructured materials, inspired by the topography of certain insect wings, has provided a potential pathway toward drug-free antibacterial surfaces, which may be vital in the ongoing battle against antimicrobial resistance. However, to produce viable antibacterial nanostructured surfaces, we must first understand the bactericidal mechanism of action and how to optimize them to kill the widest range of microorganisms. This review discusses the parameters of nanostructured surfaces that have been shown to influence their bactericidal efficiency and highlights the highly variable nature of many of the findings. A large-scale analysis of the literature is also presented, which further shows a lack of clarity in what is understood about the factors influencing bactericidal efficiency. The potential reasons for the ambiguity, including how the killing effect may be a result of multiple factors and issues with nonstandardized testing of the antibacterial properties of nanostructured surfaces, are then discussed. Finally, a standard method for testing of antimicrobial killing is proposed that will allow comparison between studies and enable a deeper understanding about nanostructured surfaces and how to optimize their bactericidal efficiency.

## Introduction

The formation of bacterial biofilms on medical devices is a leading cause of healthcare-associated infections (HCAIs), which often become chronic and require intensive courses of antibiotics to treat. Given that many of our current antibiotic treatments are failing, it is of utmost importance that we begin to look for other routes to prevent the formation of the mature biofilms which lead to infection. One way to effectively prevent the formation of biofilms on medical devices is to confer their surfaces with antibacterial properties. Such antibacterial surfaces act to prevent the proliferation of bacteria and are categorized as either antifouling^1^ (preventing the initial attachment of bacteria) or bactericidal^2^ (direct killing of bacteria upon contact). This review will focus on the latter.

Initial designs of bactericidal surfaces used chemical methods alone, such as embedding silver nanoparticles^3−7^ or coating with antimicrobial compounds,^8−10^ to achieve their bacteria-killing effect. These approaches have proven to be successful; however, they come with associated issues such as environmental toxicity and decreasing effectiveness over time as the concentration decreases due to degradation of the active compound.^2^ The growing impact of antimicrobial resistance must be also considered when using chemical methods to kill bacteria.^11−13^ Consequently, alternative methods of producing bactericidal surfaces are highly desirable.

The past decade has seen an increase in the development of surfaces that utilize mechano-physical methods to create an antibacterial effect. These surfaces were originally inspired by biological structures such as lotus leaves,^14−16^ shark skin,^17,18^ cicada wings^19−24^ and dragonfly wings.^25−28^ The microscale structures present on the lotus leaf and shark skin create a superhydrophobic surface that exhibits good antifouling properties by creating an unfavorable surface for bacteria to attach to.^15,18^ In contrast, the nanoscale structures on the wings of the cicada and dragonfly are capable of killing bacteria upon contact,^19^ creating a bactericidal effect. It was proposed that this killing process occurs purely as a result of the mechanical interaction between the bacteria and the surface nanopillars, creating the possibility of drug-free bactericidal surfaces.

**Figure 1:**
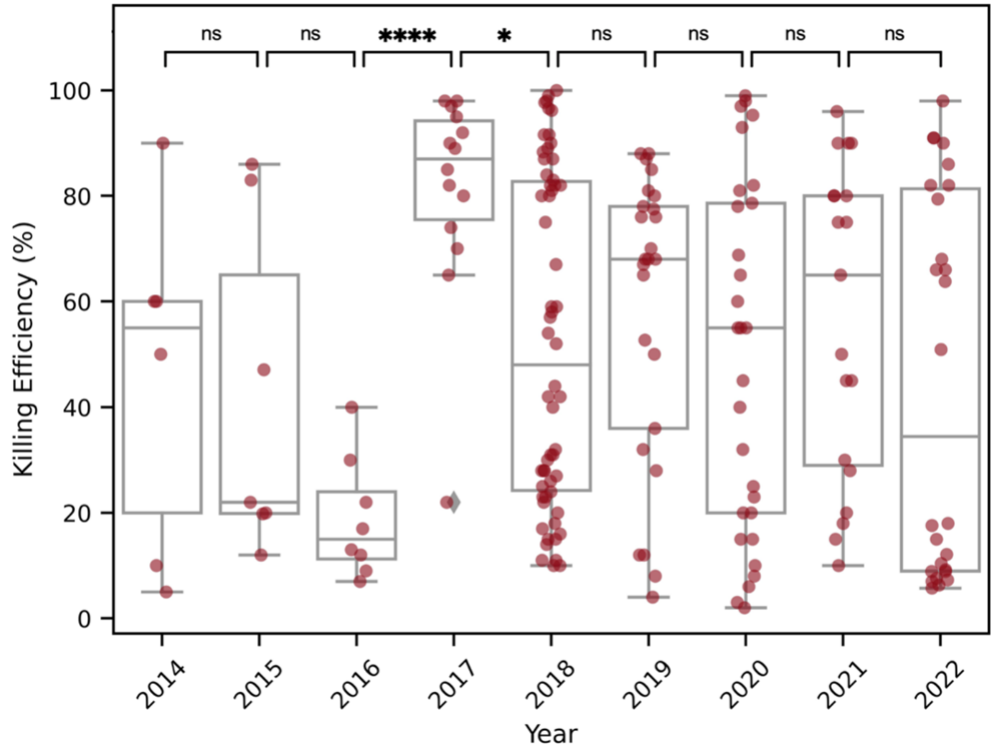
Reported bactericidal efficiencies of NSS from studies in the literature. Only studies that reported the killing efficiency as a percentage of dead bacteria attached to the surface were included. Data provided by refs (21, 23, 27−42, 44−51, and 54−80). Statistical analysis was performed in the software GraphPad Prism 9, using a one-way ANOVA (ns = not significant, * *p* < 0.05, **** *p* < 0.0001).

Taking inspiration from these natural biological nanostructures has paved the way for a new class of antibacterial surface technology that acts through a mechano-physical mechanism, negating our reliance on chemicals or drugs. To date, these antibacterial nanostructured surfaces (NSS) have been fabricated from a wide range of materials including silicon,^25,29−33^ diamond,^34,35^ metals (e.g., gold,^36,37^ stainless steel,^38^ ZnO^39−41^ and titanium^42−47^), and polymers (e.g., PMMA,^48−50^ PET,^51,52^ PEEK^53^ and PS^54^). The fabrication methods for these synthetic NSS often allow close control over the parameters that define the NSS (the height, spacing, and diameter of the nanofeatures) and lead to bactericidal efficiencies that frequently exceed those of natural NSS, such as the cicada wing. This is an indication that the natural surfaces do not necessarily have the optimal parameters for bacterial killing, and that by engineering NSS to have different feature parameters, it may be possible to push this efficiency to a range where it would become useful as a new antibacterial technology. However, as Figure 1 shows, the bactericidal efficiency of the NSS that have been investigated in the literature has not improved appreciably year-on-year. The only significant increase came in 2017, when there was also an increase in the number of studies on antibacterial NSS. This highlights the lack of understanding of the factors that influence the bacteria-killing ability of NSS and shows work is still required in order to produce NSS that could become a viable antibacterial technology. This review will explore these factors with the aim of outlining the work that is still required to optimize the effectiveness of antibacterial NSS. It will also highlight the need for more rigorous testing regimes for antibacterial NSS, which would greatly aid further research and understanding.

## Mechanobactericidal Mechanisms

Recent reviews have explored the potential bactericidal mechanisms of NSS in great detail,^81,82^ so only a short summary will be presented here. One of the first theories to explain the bactericidal mechanism of NSS used a theoretical, biophysical approach to explain the interactions between the bacterial cell wall and the nanostructures. In this theory, the bacterial cell envelope was described as a thin elastic sheet that experiences an increase in surface area as it comes into contact with the surface nanostructures.^83^ At a certain degree of stretching, the cell membrane is thought to rupture, resulting in a loss of turgor pressure and death of the bacterium. This theory predicts that the optimal NSS for killing bacteria would have features that create the greatest amount of curvature in the bacterial envelope.^83^ There is now growing evidence that other factors may also be involved in the bactericidal mechanism of NSS. Bandara et al. reported that strong adhesion between the nanostructures and the bacteria can lead to large shear forces acting on the cell wall due to the movement of the bacteria as they grow and divide.^27^ This implies that surface topographies that create higher adhesion forces would lead to an enhancement in killing. For flexible NSS, it was also recently demonstrated that the elastic forces from the bending and subsequent restoration of the nanofeatures could contribute to the stretching of the bacterial envelope and so the amount of killing.^79^

The physiological response of the bacteria to the nanostructures must also be considered when investigating the mechanobactericidal mechanisms. The bacterial cell wall undergoes stress-stiffening in response to changes in osmotic pressure,^84,85^ which increases the Young’s modulus of the cell wall, making it harder to rupture. Jenkins et al. discovered that while deformation of the *Escherichia coli* membrane was observed on TiO_2_ NSS, little/no mechanical rupture or cell lysis occurred. Instead, they proposed that bacteria produce reactive oxygen species (ROS), such as H_2_O_2_, in response to the stress associated with the interaction with NSS, which could contribute to cell death.^43^ Very recently, it has been suggested that the mechanical damage sustained by bacteria as a result of the interaction with NSS is not sufficient to kill them. Instead, it was proposed that the injury leads to an apoptosis-like response from the cells that ultimately causes their death and that accumulated ROS can induce this response, even once the mechanical stress from the nanofeatures has been removed.^86^

These recent works demonstrate that the processes involved in bacterial death on NSS are still not fully understood and that the picture may be much more complex than first thought, especially when considering the biological mechanisms that may be involved. The nanoscale nature of the interactions between the NSS and the bacterium makes direct visualization of the bactericidal mechanism extremely challenging; therefore, much of the consensus on how bacteria are killed is inferred from investigations into the role that the surface parameters play in the bactericidal efficiency of NSS.

## NSS Parameters Influencing Bactericidal Efficiency

**Figure 2:**
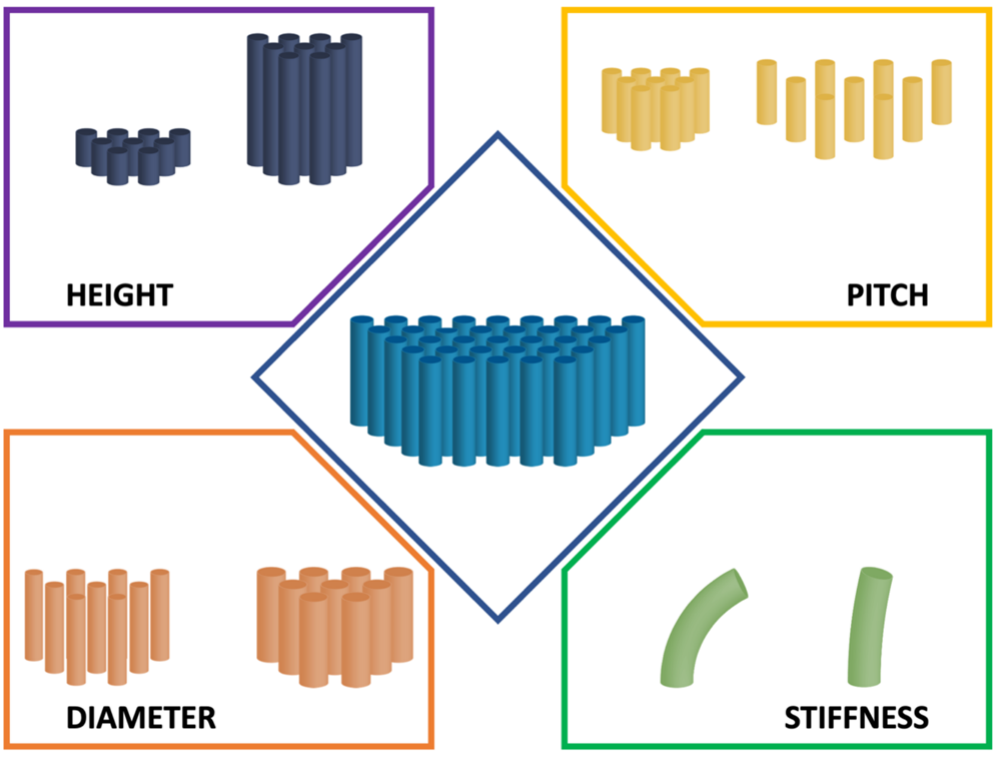
NSS parameters that have been shown to influence the bactericidal efficiency include the nanofeature height, diameter, pitch (spacing) and the stiffness/Young’s modulus.

Given that the bactericidal nature of NSS is due to mechanical effects, the physical interaction of the bacteria with the nanofeatures is a key factor that determines their killing efficacy. These interactions depend on both the characteristics of the feature surface and the bacterium. The characteristics of the surface that have been reported to influence bactericidal efficiency include the height,^52,72,87^ diameter,^48,51,88,89^ pitch (spacing)^48,51,52,63,87,88,90^ of the nanofeatures, and more recently the material stiffness/Young’s modulus^61,79,91^ (illustrated in Figure 2).

## Nanofeature Pitch/Density

Theoretical models predict that for nanofeatures to exert enough stress on the bacterial membrane to initiate a bactericidal effect, their spacing (pitch) must not exceed the diameter of the bacterium.^87^ Surfaces with features which are more widely spaced than the bacterial cell size tend to cause the bacteria to align in the grooves between the nanofeatures, which does not lead to a bactericidal effect.^92,93^*E. coli* and *Bacillus subtilis*, model Gram-negative (gram −ve) and Gram-positive (gram +ve) bacteria, both have typical diameters of ∼1 μm,^94,95^ and so NSS with a feature spacing >1 μm would not be expected to have bactericidal properties. Numerous studies have suggested that a closer spacing of nanofeatures will result in a higher bactericidal efficiency,^21,48,51,79,90,96^ which is often reconciled with the claim that a higher feature density should lead to a greater amount of stress being imparted on the bacterial membrane.

However, there are some contrasting reports, both from theoretical models^88^ and from experimental data.^32,52,63^ Assuming the “biophysical” model of bacterial death on NSS, Li predicted^90^ that the degree of stretching of the bacterial membrane would increase with increasing spatial density (decreasing spacing) of nanofeatures, up to 40 features/μm^2^ when the effect plateaus. However, subsequent models predict that a greater spacing of the nanofeatures will lead to more stress on the membrane (if the requirement is met that the spacing is not wider than the bacteria) and should therefore lead to a higher bactericidal efficiency.^88^ Experimentally, it was shown that decreasing the spacing of PET nanocones from 500 to 200 nm increased their ability to kill *E. coli*, with up to 16% and 30% killed on each surface, respectively,^51^ which agrees with the theoretical prediction by Li. Similarly, PMMA NSS have been fabricated using nanoimprint lithography with feature spacings of 100, 130, and 380 nm. Here the percentage of *E. coli* killed increased with decreasing feature spacing, with 22% killed on the 100 nm spacing and only 12% killed on the 380 nm spacing.^48^ In contrast, Wu et al. found that the optimal density of polymer nanofeatures to kill *Staphylococcus aureus* was ∼40 features/μm^2^ (98–100% killing) and that there was a significant reduction in killing ability for <20 features/μm^2^ (26-31%) and >60 features/μm^2^ (23–31%).^63^ However, here the height of the features was not kept constant. Recently, the killing effect of nylon NSS with a nanofeature spacing of 60, 100, and 200 nm and fixed aspect ratio, was assessed against *Pseudomonas aeruginosa* and *S. aureus*.^52^ It was found that for *P. aeruginosa*, the killing efficiency increased with decreasing spacing (from 35% to 90%), whereas for *S. aureus*, the killing efficiency was the highest for both the smallest and largest spacing.

While there are some varied conclusions on the optimal feature spacing, the consensus from the literature is that closer spaced nanofeatures will lead to a higher bactericidal efficiency for nanostructured surfaces, up to a point. The key basis for the link between the feature spacing and the bactericidal ability is the number of contact points between the surface and the bacteria. As the feature spacing reduces, the number of contact points with the cell wall increases, thus enhancing the stress imparted. As the spacing continues to reduce, the topography tends toward a flat plane, and the overall stress on the membrane reduces.

## Nanofeature Diameter, Height and Aspect Ratio

As with the nanofeature spacing, changing the diameter of the features is expected to have a direct effect on the amount of stress experienced by the bacterial membrane. However, again, there are conflicting results from theoretical models based on the “biophysical” mechanism. Li postulated that the degree of membrane stretching would increase with increasing nanofeature radius, although the calculations suggest that the effect is small above diameters of 20 nm.^61^ Other recent modeling suggested that decreasing feature radius from 30 to 10 nm could increase the strain on the bacterial envelope by ∼25%.^89^

Looking at differences in diameter experimentally, Hazell et al. found that PET nanocones with narrower tips killed a greater proportion of *E. coli* – a diameter of 300 nm killed 10% of attached cells, whereas a diameter of 20 nm killed 20%.^51^ A study on PMMA NSS also found that reducing the width of the features from 215 to 70 nm increased the killing of *E. coli* from 12% to 22%.^48^ These experimental studies suggest that reducing the radius of the features increases the bactericidal efficiency of the NSS.

It has been suggested that nanofeatures have a “minimum” height requirement to trigger an antibacterial effect. Watson et al. showed through simple modeling how the nanofeatures must be sufficiently long to allow the bacteria to be pulled down, causing enough membrane deformation to result in cell death.^87^ Similar results have also been gathered through various experimental studies. Surfaces covered in 150 nm tall polycarbonate nanofeatures were shown to kill 3% of *E. coli*, whereas the surfaces with features >150 nm tall were able to kill ∼90% of the attached bacteria.^72^ Linklater et al. reported seeing minimal envelope disturbance for *S. aureus* and *P. aeruginosa* on polymer NSS with 30 nm high features, but significant damage was observed on surfaces with 120 and 220 nm tall features.^52^ It appears that while increased height does increase bactericidal efficiency, once the critical height of the nanofeatures is reached, any further increase does not have a substantial impact on killing. This could be because the bacteria only interact with the top of the nanostructures, and so nanofeatures with different heights will “look the same” to the cell, providing the pitch and diameter also remain the same.

The etching techniques commonly used to fabricate synthetic NSS (such as reactive ion or plasma etching)^25,32,34,35,51,53,67,97^ often lead to the height and diameter of the nanofeatures being simultaneously varied and so becoming entangled. Therefore, it is common to refer to the aspect ratio (the ratio of the height and diameter) of the nanofeatures. Although, as noted by Cui et al.,^72^ nanofeatures that are not cylindrical will have varying aspect ratios depending on where the diameter is measured. Michalska et al. noted that the antibacterial properties of high aspect ratio black silicon nanofeatures exceeded those of the lower aspect ratio counterparts and suggested that this could be due to a different killing mechanism for the two types of surface.^33^ For ultrahigh aspect ratio features (>1000), clustering of the tips creates a more favorable surface for the bacteria and has been shown to lead to a reduction in the bactericidal efficiency.^61^

## Nanofeature Mechanical Properties

Until recently, the effect of the mechanical properties of the NSS had been overlooked as a factor that could impact the bactericidal efficiency. The NSS described in the literature are made from a range of different materials that have large differences in mechanical properties. For example, a common organic material used to make NSS is PMMA, which has a Young’s modulus of ∼3 GPa.^98^ Whereas silicon, an inorganic material that has been widely used for NSS, has a Young’s modulus >130 GPa.^99^ Such a wide range of mechanical properties could be expected to affect the interaction between the bacteria and the nanofeatures.

In theoretical studies, the nanofeatures are often assumed to be much stiffer than the bacterial membrane and therefore unlikely to deform as the bacteria adsorb/attach to the surface. This is the basis of the idea that the bacterial membrane is “stretched” to breaking point over the nanofeatures. However, there is now increasing evidence to the contrary. Ivanova et al. recently reported that an increase in flexibility of high-aspect ratio silicon nanofeatures was responsible for an increase in bactericidal efficiency against both *P. aeruginosa* and *S. aureus*.^91^ They found that increasing the height of the nanofeatures led to an increase in their flexibility and the amount of elastic energy stored in them when bent as a result of the interaction with the bacteria. They concluded that this additional elastic energy stored in the nanofeatures could lead to extra stress on the bacterial membrane. Similarly, it was reported that superhigh aspect ratio carbon nanotubes, which were able to store a greater amount of elastic energy, killed a higher percentage of both *P. aeruginosa* and *S. aureus*.^61^ Very recently, Lohmann et al. created NSS from a range of UV-cured resins with differing Young’s modulus. Unlike the other studies on the flexibility of the nanofeatures, here the surface topography is kept constant, and the feature stiffness changed between 208 MPa and 4 GPa.^79^ When testing the killing effect of these NSS against *E. coli*, they found that only the features with a stiffness ≥1.3 GPa were able to kill significantly more bacteria than the flat control surfaces. They concluded that, due to their increased stiffness, these surfaces were able to exert strong shear forces on the bacterial membrane that contributed to the overall stress, increasing the chance of rupture.

These early reports suggest there could be a link between the mechanical properties of NSS and the bactericidal efficiency. They are also counter to the assumption from the theoretical models that the nanofeatures must be straight and rigid in order to stretch the bacterial membrane and cause cell death.

## Surface Wettability

Given that the initial attachment of the bacteria to the surface must play a key role in the bactericidal action of NSS, it is important to consider the factors which may influence this. Wettability (determined by water contact angle (WCA) measurements) is known to be an important parameter in the early attachment of bacteria to surfaces;^100−102^ however, the exact relationship between hydrophobicity and bacterial attachment is still contested.^101,103−105^ For example, it was shown that *E. coli* had the highest levels of adhesion on moderately hydrophobic surfaces (WCA = 95°) and the lowest on both hydrophilic (WCA < 30°) and superhydrophobic (WCA > 120°) surfaces.^106^ Antifouling surfaces typically prevent the attachment of bacteria through superhydrophobic properties, as is the case with many microstructured surfaces.^14,107−109^

For bactericidal NSS, there have been several studies that investigated the potential link between the surface hydrophobicity and the bacteria-killing effect. Boinovich et al. found that *E. coli* were killed to a higher degree on superhydrophilic titanium NSS compared to superhydrophobic surfaces,^110^ reporting that the attractive interaction between the bacteria and the hydrophilic surface enhanced the damage caused by the nanofeatures. More recently, Valiei et al. demonstrated that hydrophilic surfaces were more effective at killing *P. aeruginosa*, but only when the NSS had just been dried.^111,112^ They suggest that a combination of the hydrophilic properties of the surfaces and the capillary forces which arise during evaporation drive the bactericidal effect. In contrast, Linklater et al. created NSS from acrylic that had been chemically modified with fluoroalkyl groups to render it hydrophobic or poly(ethylene oxide) (PEO) chains to render it hydrophilic.^52^ They found that the NSS made from the hydrophobic acrylic showed enhanced bactericidal efficacy against both *P. aeruginosa* and *S. aureus* compared to the hydrophilic acrylic.

Once again, however, the wettability of NSS often becomes entangled with other parameters, as the nanoscale roughness of these surfaces generally confers them with their superhydrophobic properties.^113,114^ It is possible to negate this by chemically modifying the NSS to change hydrophobicity, which has been demonstrated in the past,^52,112^ but considerations must be taken to ensure that these chemicals themselves are not contributing to the bactericidal effect. These considerations mean that a clear link between surface hydrophobicity and the bactericidal efficiency is yet to be established.

## Literature Analysis

It is clear that there is no consensus on the optimal nanofeature parameters to enhance bactericidal efficiency. Often, the theoretical models come to differing conclusions to each other and to the experimental data, which raises questions about the validity of the models and assumptions in these studies. Most of these models base their assumptions on the simplified stretching model of cell death and do not consider any biological processes, such as the production of reactive oxygen species that could occur as a result of the interaction. Given that it seems increasingly likely that biological processes do play a role,^43,86^ it may be that these simple models no longer provide enough information to make accurate predictions.

**Figure 3:**
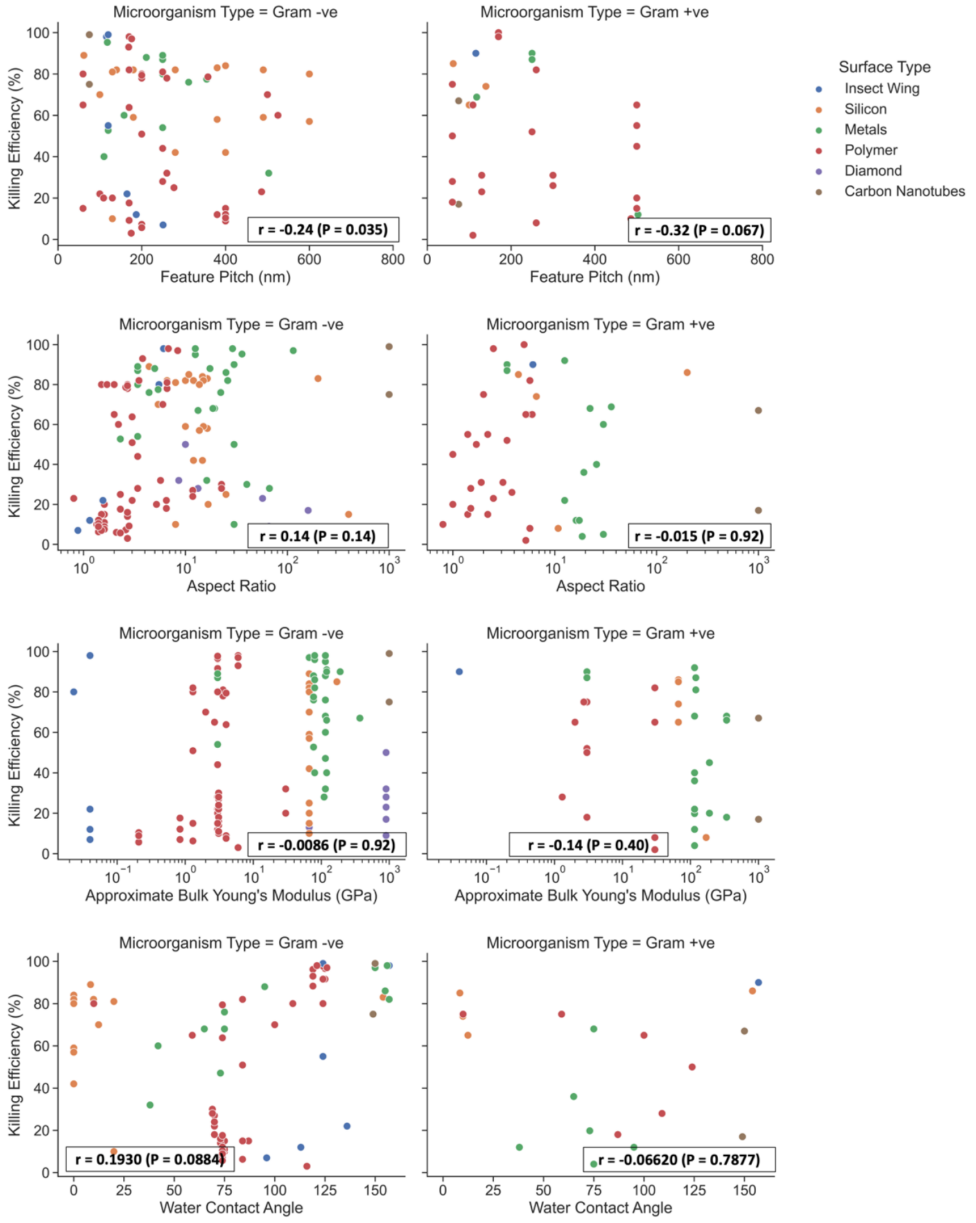
Summary of the bactericidal efficiency of NSS reported in the literature versus the feature pitch, aspect ratio (height/diameter), approximate Young’s modulus and water contact angle. In cases where Young’s modulus values were not given, an approximation was made based on the bulk value of the material. Only studies which reported the killing efficiency as a percentage of dead bacteria attached to the surface were included. Correlations between the parameters and the killing efficiency was assessed using the data analysis software GraphPad Prism with the Pearson correlation coefficient, *r*, along with the *P*-value to test significance (α = 0.05). Sample sizes (*n*) for each test are pitch (gram −ve) = 78, pitch (gram +ve) = 34, aspect ratio (gram −ve) = 116, aspect ratio (gram +ve) = 45, Young’s modulus (gram −ve) = 124, Young’s modulus (gram +ve) = 37, contact angle (gram −ve) = 79, contact angle (gram +ve) = 19. Data provided by refs (20, 21, 23, 25, 27−30, 32−42, 44−51, 53−55, 57−80, and 96).

To explore the factors that influence the bactericidal efficiency of NSS further, a meta-analysis of the literature was performed. The 580 reports that cited the original work by Ivanova et al.^19^ were analyzed, with review articles and works which focused on chemical-based bactericidal surfaces removed. Of the remaining 57 articles, 49 reported on the bactericidal efficiency of an NSS and are summarized in Figure 3. For all the nanofeature parameters, there was only a small correlation, if any, found between the parameter and the resulting bactericidal efficiency. Reducing the pitch of the nanofeatures significantly correlated with an increase in killing efficiency (for gram −ve bacteria). However, for the rest of the parameters, there was no significant correlation to the resulting bactericidal efficiency.

**Figure 4:**
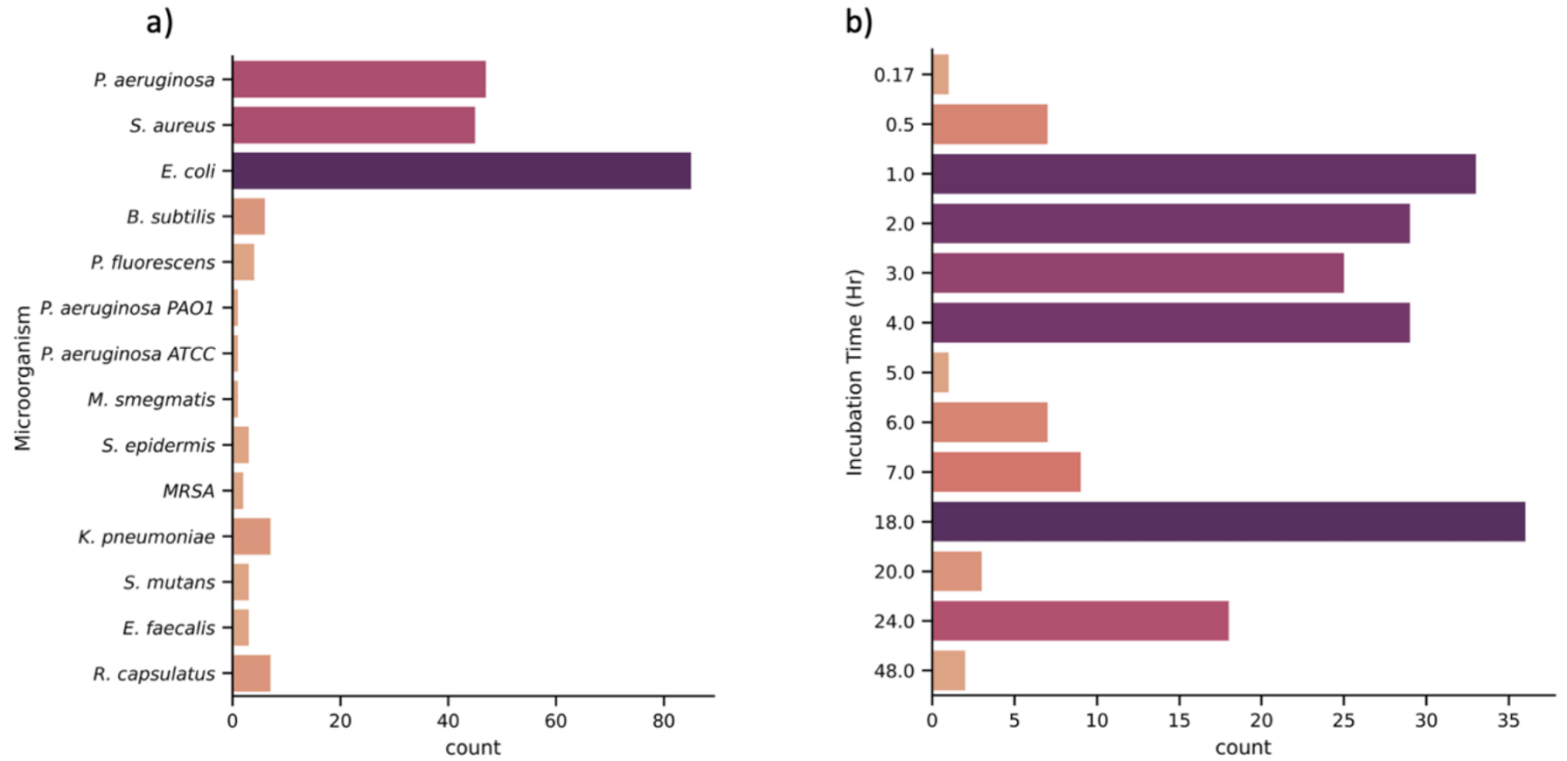
Histograms of the testing conditions of the NSS taken from the literature. (a) The number of instances of each microorganism being tested in studies and (b) the number of instances of each incubation time. Data provided by refs (20, 21, 23, 25, 27−30, 32−42, 44−51, 53−55, 57−79, and 96).

A key reason for this is that, for most of these studies, more than one of these surface parameters was varied at a time.^21,33,37,48,51,67,96^ As previously discussed, it is likely that this is due to the etching-based fabrication techniques used to create these surfaces;^25,32,34,35,51,53,67,97^ however, this makes it difficult to interrogate the effects of each and determine which factor has the biggest impact on the bactericidal efficiency. Furthermore, as Figure 4 shows, the method of determining the bactericidal efficiency of the NSS also lacked consistency across the different studies, making it difficult to compare the results. It is well established that Gram-negative bacteria are more susceptible than Gram-positive bacteria to killing by NSS due to their thinner cell envelope.^20,25^ Many investigations in the literature tested the NSS against *E. coli* (gram −ve),^25,27,30,33,38,39,41,42^*P. aeruginosa* (gram −ve)^20,23,25,28,29,50,50,55^ or *S. aureus* (gram +ve).^25,29,32,50,74−76,78^ However, many studies also use a range of different bacterial species^38,42,49,51,62^ (illustrated by Figure 4a), and while this is useful to assess the breadth of organisms that the NSS is effective against, comparisons between the studies become difficult. Although work has been done to establish that the shape of the bacterium does not play a role in the bactericidal efficiency,^20^ there is some evidence that the optimal feature parameters may vary between different bacterial species, possibly due to the differences in cell wall structure requiring different levels of stress to initiate cell death.^33,49,52,67^ The presence of cell wall structures may also affect the bactericidal efficiency. Jindai et al. demonstrated that flagella can become tangled in nanofeatures, causing the bacteria to become trapped near the surface and be damaged by the structures more frequently.^115^

The growth phase of the bacteria is a factor often overlooked in the assessment of NSS. Truong et al. showed that bacteria in different stages of growth are killed to different degrees by NSS^57^ and so comparisons between studies that have grown bacteria to different phases (i.e., midexponential or stationary) are potentially invalid.

A greater problem is the variation in the time that the bacteria are incubated on the NSS between the different studies. As Figure 4b shows, there is no consistency in incubation time, with many studies exposing the bacteria to the NSS for either 1, 2, 3, 4, or 18 h. Given that these surfaces are bactericidal due to a contact-killing action, it is highly likely that exposing the bacteria to the nanofeatures for longer time periods will lead to an enhancement in the killing efficiency.^56,59^ Additionally, there is no consistency between the studies as to whether the bacteria are incubated on the surfaces in growth media (i.e., Luria Broth (LB) or Tryptic Soy Broth (TSB)) or buffered solution (i.e., PBS or NaCl solution). This will be another source of variation as the bacteria in the media will be actively growing and dividing,^116^ whereas those in buffer will not be, due to a lack of nutrients.^117^ It therefore becomes very difficult to compare the results from studies that use different incubation times and conditions.

## Conclusions and Outlook

To make progress in the quest to produce viable antibacterial nanostructured surfaces (NSS), we must understand how to optimize the NSS to achieve the highest bactericidal efficiency. In the decade since the discovery of the antibacterial effects of NSS, there has been significant research highlighting new nanostructured materials with antimicrobial effects. However, progress toward increasing their bactericidal efficiency has been hindered.

The data from the literature show no clear link between any of the individual surface parameters and the bactericidal efficiency of the NSS. This could suggest that there is no “one-size-fits-all” solution to creating effective antibacterial NSS and that the maximum bactericidal efficiency is determined by a complex combination of topographical parameters, material properties, and the test organism morphology and physiology. For example, the optimal spacing of nanofeatures will likely be different for different microorganisms,^52^ as the variation in their shape and size will change the number and distribution of the contact points. This would mean that the surface parameters would have to be tuned according to the main target microorganism and the property requirements of the material. However, the lack of clarity in the literature has also been exacerbated by the inconsistency in the way antibacterial properties of NSS are tested. Many studies vary multiple feature parameters simultaneously, which makes determining what factors have the biggest impact on bactericidal efficiency challenging. Additionally, there is currently no standardized testing method for determining the bactericidal efficiency of NSS, which has resulted in the use of a wide range of bacteria, incubation times, and conditions when testing NSS. Again, this inconsistency in the assessment of NSS prevents valid comparison of the results between different studies.

As Michalska et al. and Hawi et al. have recently stated,^81,118^ it may be necessary for the field to adopt a more standardized testing approach for determining the antibacterial properties of NSS, in order to allow more progress in the optimization of the bactericidal efficiency of NSS and also in learning more about the bactericidal mechanism. For example, the ISO standard 22196:2011, for the “measurement of antibacterial activity on plastics and other non-porous surfaces”, requires testing surfaces against both *E. coli* and *S. aureus*, incubating on both test and control surfaces in nutrient broth for 24 h, before taking colony forming unit counts to assess the bacterial survival.^119^ While this method is not designed to test the efficacy of NSS, using a similar approach would give more consistency and allow comparisons between studies in the literature.

**Figure 5:**
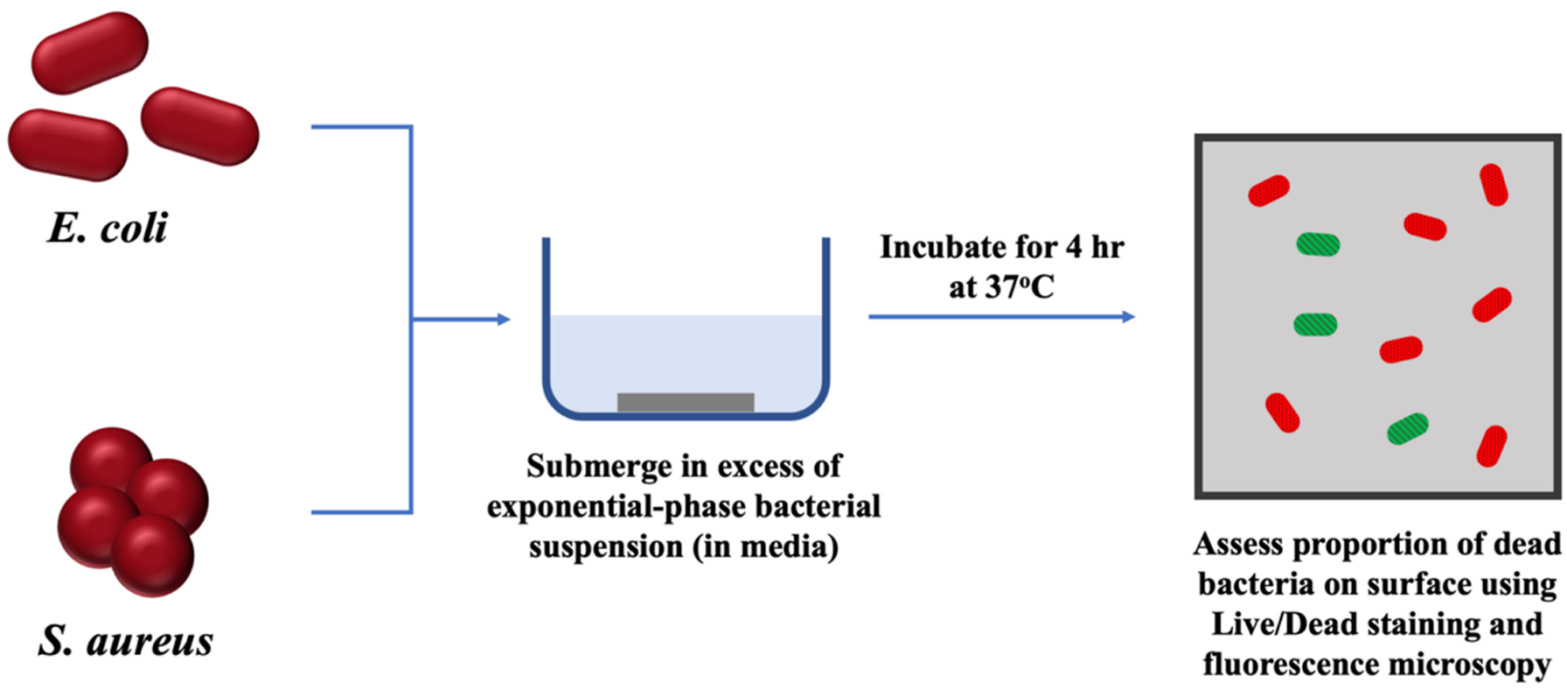
Proposed standardized method for the assessment of the bactericidal efficiency of NSS. Each NSS should be tested against exponential-phase lab strains of *E. coli* and *S. aureus* as a minimum. The surfaces should be incubated in an excess of bacterial suspension for 4 h at 37 °C. The proportion of dead bacteria should be assessed by fluorescence microscopy using a Live/Dead stain.

We propose that to assess the bactericidal efficiency of any new NSS that the following approach be taken (illustrated in Figure 5). (1) All NSS should be tested against at least *E. coli* MG1655 and *S. aureus* RN4220, which are lab strains of two common pathogenic Gram-negative and Gram-positive bacteria, respectively. By using these as reference organisms, more would be learned by testing against other strains. (2) Immerse the NSS in an excess of exponential phase bacteria suspended in nutrient broth. This is to ensure that no effects from the water contact line impact the killing, as has previously been reported,^111^ and to allow the bacteria to continue with their normal physiological process, as these may contribute to the killing mechanism. It is important to characterize the growth cycle of the bacterial strains tested as midexponential phase can occur at different growth times for each strain. (3) Incubate the bacteria on the surfaces for 4 h. This time will be sufficient for the majority of the bacteria to interact with the NSS but avoids issues with the bacteria proliferating to the point that the nanostructures are inaccessible for further interactions. Given the contact-killing nature of these surfaces, it is important for the maximum amount of the surface to be available for interaction. (4) Assess the proportion of dead bacteria on the surface with a Live/Dead stain, such as the BacLight Live/Dead bacterial viability kit, in combination with fluorescence microscopy. While there are some noted limitations of this technique,^120,121^ it is currently the most commonly used method of assessing the killing by NSS, therefore adoption of the technique would be straightforward.

A more rigorous and systematic approach to testing the factors that influence the antibacterial effect of nanostructured surfaces will be required in the future to fully understand the processes involved in killing and to optimize the feature parameters to kill a wide range of pathogens. A concerted effort by the field when creating and testing nanostructured surfaces could allow great strides to be made toward their use in clinical settings as a viable antimicrobial technology.
